# Current concepts review: Fractures of the patella

**DOI:** 10.3205/iprs000080

**Published:** 2016-01-18

**Authors:** Clemens Gwinner, Sven Märdian, Philipp Schwabe, Klaus-D. Schaser, Björn Dirk Krapohl, Tobias M. Jung

**Affiliations:** 1 Center for Musculoskeletal Surgery, Charité – University Medicine Berlin, Germany; 2 Department of Orthopaedics and Trauma Surgery – University Hospital Dresden, Germany; 3 Department of Plastic and Hand Surgery, St. Marien-Krankenhaus Berlin, Germany

**Keywords:** patellar fracture, patellectomy, conservative treatment, tension band, biomechanics

## Abstract

Fractures of the patella account for about 1% of all skeletal injuries and can lead to profound impairment due to its crucial function in the extensor mechanism of the knee. Diagnosis is based on the injury mechanism, physical examination and radiological findings. While the clinical diagnosis is often distinct, there are numerous treatment options available. The type of treatment as well as the optimum timing of surgical intervention depends on the underlying fracture type, the associated soft tissue damage, patient factors (i.e. age, bone quality, activity level and compliance) and the stability of the extensor mechanism. Regardless of the treatment method an early rehabilitation is recommended in order to avoid contractures of the knee joint capsule and cartilage degeneration.

For non-displaced and dislocated non-comminuted transverse patellar fractures (2-part) modified anterior tension band wiring is the treatment of choice and can be combined – due to its biomechanical superiority – with cannulated screw fixation. In severe comminuted fractures, open reduction and fixation with small fragment screws or new angular stable plates for anatomic restoration of the retropatellar surface and extension mechanism results in best outcome. Additional circular cerclage wiring using either typical metal cerclage wires or resorbable PDS/non-resorbable FiberWires increases fixation stability and decreases risk for re-dislocation. Distal avulsion fractures should be fixed with small fragment screws and should be protected by a transtibial McLaughlin cerclage. Partial or complete patellectomy should be regarded only as a very rare salvage operation due to its severe functional impairment.

## Zusammenfassung

Obwohl Patellafrakturen lediglich für ca. 1% aller knöchernen Verletzungen verantwortlich sind, können sie – aufgrund ihrer Bedeutung für den Streckapparat – zu ausgeprägten Funktionseinschränkungen führen. Die Diagnose wird in Zusammenschau des Unfallmechanismus sowie der klinischen und radiologischen Untersuchung gestellt. Auch wenn sich auf diese Weise die Mehrzahl der Frakturen nachweisen lässte, steht dieser eine Vielzahl unterschiedlicher Behandlungsmethoden gegenüber. Die Auswahl hängt vom Frakturtyp, Weichteilschaden, individuellen Patientencharakteristika (z.B. Alter, Knochenqualität, Aktivitätsniveau und Compliance) und der Integrität des Streckapparates ab. Ziel jeder Behandlung muss es sein, eine frühfunktionelle Nachbehandlung zu ermöglichen, um Folgeschäden wie Bewegungseinschränkungen zu minimieren.

Die modifizierte Zuggurtungsosteosynthese ist mittlerweile Methode der Wahl und kann – aufgrund ihrer biomechanischen Überlegenheit – mit einer kannülierten Schraubenosteosynthese kombiniert werden. Bei Trümmerfrakturen sollte zudem eine Refixation der einzelnen Fragmente mittels Kleinfragmentschrauben angestrebt werden. Die zusätzliche Anlage einer Tonnencerclage, entweder als Drahtcerclage oder als resorbierbare PDS- bzw. nicht-resorbierbare FiberWire-Cerclage erhöht die Konstruktstabilität und verringert das Risiko einer Re-Dislokation. Distale Patellafrakturen bzw. Avulsionsfrakturen der Patellasehne sollten mittels Kleinfragmentschrauben refixiert werden und zusätzlich durch eine McLaughlin-Cerclage gesichert werden. Eine partielle oder komplette Entfernung der Patella sollte – augrund der einheitlich schlechten Ergebnisse – unbedingt vermieden werden.

## Etiology and epidemiology

Fractures of the patella are serious injuries with a broad range of subtypes. These injuries account for about 1% of all skeletal injuries and are most prevalent within the age group of 20–50 years [84]. Epidemiologic studies demonstrated that the incidence in men is twice as high as in women [9], [52]. Because of the subcutaneous anterior location, the biomechanical function and the high level of force transmission during extension and flexion, stable reconstruction of patellar fractures continues to represent a major surgical challenge. The majority of cases are caused by direct injury mechanism [65]. The resulting fracture type depends on the trauma mechanism (i.e. direct or indirect), the energy transmitted to the bone and the bone quality. The most common fracture pattern is a simple 2-part diversion caused by a direct blow (i.e. dashboard injury). As a result of the bony lesion the extensor mechanism of the knee joint can become insufficient. The degree of the insufficiency depends among other factors on accompanying damage to the reserve extensor mechanisms. Additional injuries to the adjacent bones are rare but can affect the articular surface of the distal femur. The most frequent indirect mechanism is a fall on the feet with eccentrically contraction of the quadriceps muscle. Depending on the velocity of the fall and the resistance of the extensor mechanism, either the patella or the adjacent tendons fail [75].

Closed fractures of the patella represent the vast majority of this injury. However, up to 7% of the cases result in open fractures [9], [80]. The underlying mechanisms of open fractures are mostly high velocity accidents. These can result in devastating soft tissue conditions with comminuted fractures as well as additional ruptures of the reserve extensor mechanism. Of note, approximately 80% of open patellar fractures are associated with multiple accompanying injuries, namely fractures of the femur or acetabulum, traumatic dislocation of the hip joint or disruption of knee ligaments [16], [80]. 

Most frequent causes are traffic accidents in 78.3%, followed by work-related accidents in 13.7% and domestic accidents in 11.4% [84]. Sports-related fractures of the patella are relatively seldom. However, acute dislocations of the patella are associated with osteochondral fractures of the patella or fractures of the lateral femoral condyle in up to 70% of cases [64]. 

Periprosthetic fractures of the patella are regarded as a typical complication of total knee arthroplasty (TKA), with an incidence of 0.68% in unresurfaced patellae and up to 21% resurfaced patellae [54], [66]. In addition, fractures to the patella are described as a rare donor site complication of patella tendon harvesting for anterior cruciate ligament reconstruction [5], [7], [68] and during reconstruction of the medial patellofemoral ligament [69], [70].

## Anatomy

The patella is the largest sesamoid bone of the human body and is embedded in the quadriceps tendon. It is one of the few bones without a periosteal surrounding. The proximal three-fourths of the patella are covered by a thick layer of cartilage, whereas the remaining distal pole is not part of the articular congruency. The adjacent quadriceps muscle consists of four muscles, of which the rectus femoris is the longest and most superficial. The deep layer of the quadriceps tendon inserts at the proximal basis of the patella whereas the superficial fibers extend over the patella itself continuously to the tibial tuberosity. 

The fascia lata spreads over the anterior surface of the knee and forms the patellar retinaculum in combination with aponeurotic fibers introduced by the lateral and medial vastus muscle. Deep transverse fibers of the joint capsule, known as the patellofemoral ligaments – radiating from the patella to the femoral epicondyles – contribute to the patellar retinaculum [72]. These fibers have a crucial function as they allow some degree of active extension even in presence of a patellar fracture (reserve extensor mechanism).

The patella ossifies between 4 and 7 years of age. During development, the patella originates from a single ossification center in most cases. Approximately 23% of the population possess up to three ossification centers which do not merge in 2% of the cases. This results in either in a bipartite or tripartite patella and is typically located at the upper patellar pole. In 50% of the cases this phenomenon is seen bilaterally. However, patellae bipartite or multi partita remain asymptomatic in most cases. 

Wiberg subdivides anatomic alterations of the patella into three types based on the size of the medial and lateral facets. In type I, medial and lateral facets are approximately equal, whereas the medial facet becomes steadily smaller in type II and III [83].

## Biomechanics

The extensor mechanism of the knee is based on a complex network of static and dynamic stabilizers that converge towards the centrally located patella. Within this construct the patella functions as a lever arm for knee extension, effectively augmenting the quadriceps force and consequently contributing to the extensor mechanism of the knee [75]. The crucial function of the extensor mechanism of the knee is to maintain the erect position as well as to realize the unassisted gait. The principal purpose of the patella is linking and displacement [40]. During flexion it is located in the groove of the femoral trochlea acting primarily as a link between the quadriceps muscle and the proximal tibia. At 45° to 60° of flexion the proximal part of the patella, which is thus covered by a thick layer of cartilage, has to withstand the most pressure [25]. Between 45° of flexion and full extension the patella increases the effective lever arm of the quadriceps by displacing the linkage between quadriceps and tibia away from the axis of knee rotation. Biomechanical studies have demonstrated that this increases the lever arm of the quadriceps by up to 30% at full extension [40].

Vice versa, the patella also plays an important role in the resistance of knee flexion [38]. It converts tensile forces into compression forces and thus decelerates knee flexion in particular during walking down stairs or downhill [3], [45]. This is also known as the “patella femoral joint reaction (PFJR)”. To fulfill its function, the patella has to withstand high forces which have been shown to be as high as 3200N equaling four to five times standard body weight [36].

It is assumed that 8 to 12 weeks are necessary to achieve bony healing of a fracture of the patella passing through approximately 100,000 cycles of flexion and extension within this period [11]. These facts reflect a high demand on any treatment strategy. 

## Diagnosis and clinical manifestation

The diagnosis of a fracture of the patella is made on the basis of the injury mechanism, physical examination and the radiological findings. It is suspected in all patients who have sustained a direct impact to the anterior knee and are unable to actively extend their knee after flexion injury or fall [75]. 

The clinical investigation usually shows in displaced fractures a visible and palpable defect between the bone fragments, local hematoma and a hemarthros that occurs quickly and symmetrically on the anterior knee as almost all patellar fractures communicate with the knee joint. The complete inability to extend the knee reveals that a tear of the medial and lateral retinaculum is present in addition to the fracture. Vice versa, the ability to extend the knee actively does not exclude a fracture sufficiently, since either the retinacula or the iliotibial band and the adductors can provide active knee extension in patellar fractures without significant displacement. In cases with the history of a dashboard injury, with a direct blow to the anterior knee or proximal tibia, an examination of both the knee and hip joint has to be obtained. Especially the integrity of the posterior cruciate ligament, the distal femur and the acetabulum must be confirmed. 

Evaluation of the soft tissue status is crucial as the degree of soft tissue damage determines the further course of the patient. In up to 25% of cases a skin abrasion is present, which can interfere with the timing of surgery as well as the surgical approach [13].

Osteochondral fractures of the patella are usually a result of shear forces caused by a patellar dislocation and occur less frequently by a direct impact trauma of the patella [33], [64], [78]. Patients rarely have an extension deficit, but the affected knee commonly presents with an acute hemarthrosis and a positive apprehension sign of the patella as described by Fairbank [21].

Biplanar radiographs of the knee joint are mandatory as they show the patella as well as the surrounding bony structures. An axial view should be avoided in the acute situation due to discomfort and secondary dislocation of the fracture. In presence of a symmetrical high or low position of the patella in relation to the contralateral side, a patella alta or baja is likely. Contrary, an asymmetrical patellar position implies a disruption of the quadriceps or patellar tendon until proven otherwise. 

In cases of comminuted fracture patterns or suspected accompanying ligamentous, meniscal or osteochondral injuries, a CT scan including multiplanar reconstructions offers exact imaging of the fracture pattern while a MRI can contribute to detect additional injuries.

## Classification

In principal, traumatic fractures of the patella are classified as transverse, vertical, comminuted, marginal or osteochondral. Transverse fractures occur horizontally across the patella and are most often due an indirect impact on the patella (i.e. falls). Vertical fractures typically run from the inferior to the superior pole and may be stable and treated conservatively. Fractures to the margins of the patella occur at the perimeter of the patella and commonly due a direct force to the side of the patella. Comminuted fractures are often seen in multiple injured patients. These cases often present with a high degree of soft tissue damage. Cramer et al. postulated that fractures with a displacement of less than 3 mm should be considered non-displaced [18]. 

**Figure 1:**
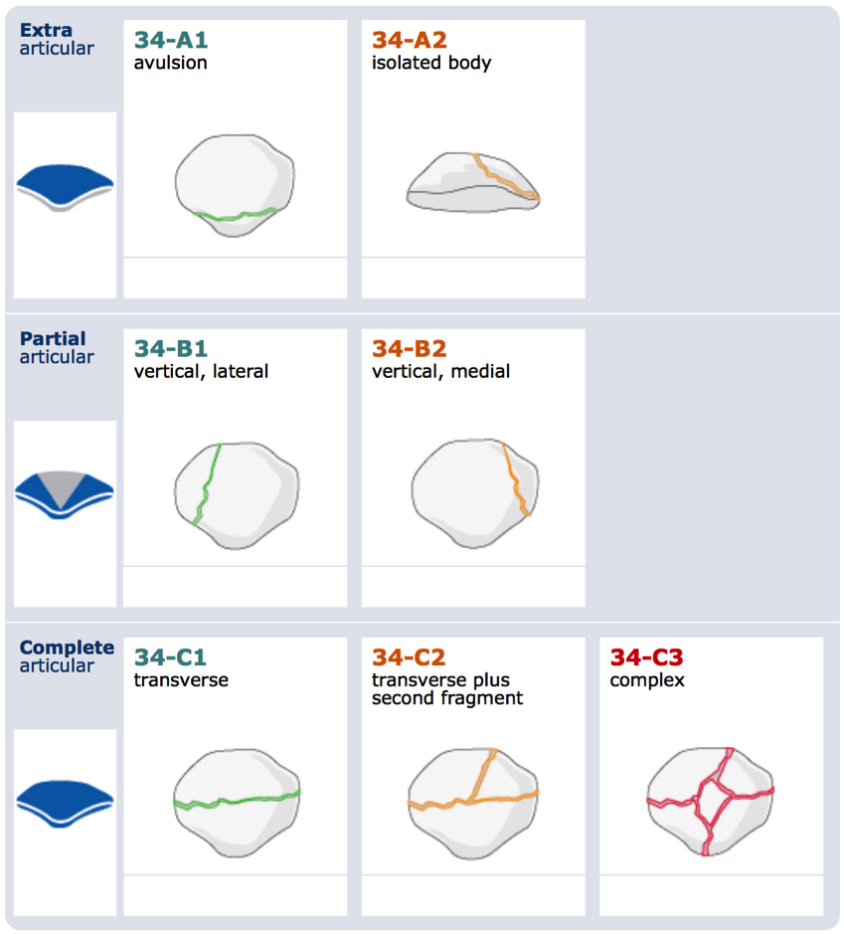
Classifications of patellar fractures according to the AO/ASIF, reprinted with permission of the AO Foundation Copyright by AO Foundation, Switzerland

The AO (Arbeitsgemeinschaft für Osteosynthesefragen) – ASIF (Association for the Study of Internal Fixation) has advocated a classification – (Figure 1 (Fig. 1)) based on the principles of the classification of long bone fractures as published by Müller et al. [60].

Patellar fractures are a diverse group of injuries and the fracture type varies considerably. To date, a useful classification system that incorporates these distinctions and thus leads the surgeon to a specific treatment concept is still missing. 

## Decision making

The most crucial consequence of a patellar fracture can be the discontinuity of the extensor mechanism of the knee joint resulting in inability of active knee extension. Furthermore, these fractures can lead to a mismatch of the patellofemoral articulation. Consequently, the goals of treatment have to aim for anatomic reduction, stable fixation with restoration of both, the articular surface and the extensor mechanism as well as the ability of early rehabilitation.

A failure of these objectives may lead to an early onset of patellofemoral osteoarthritis accompanied with impaired motion and persistent knee pain. In conclusion, the type of treatment is chosen on an individual basis and depends on the fracture pattern, extent of soft tissue damage, number and size of the fragments, congruence of the articular surface, stability of the extensor mechanism and patient’s compliance.

## Conservative treatment

**Figure 2:**
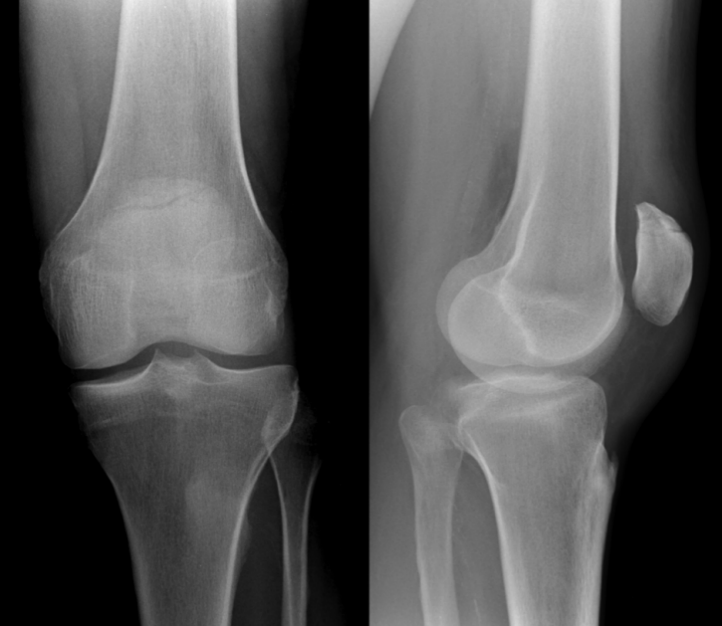
Non-displaced fracture of the proximal patellar pole, which might be suitable for conservative treatment. Notably, these cases should be tested up to 60° of flexion under image intensifier control in order to confirm a stable fracture pattern.

Stable non-displaced fractures (less than 2 mm of dislocation) are suitable for a conservative approach (Figure 2 (Fig. 2)). These cases should be tested up to 60° of flexion under image intensifier control in order to confirm a stable situation with no tendency for dislocation. Braun et al. and Böstman et al. postulated that even comminuted fractures as well as proximal or distal pole fractures can be treated non-operatively if they fit the above mentioned precondition [8], [12]. Exceptions to this rule are chondral or osteochondral fractures in which operative refixation should be obtained even if dislocation meets the mentioned criteria of conservative treatment. 

Conservative treatment should aim for early joint motion. Thus 40° of knee flexion should be obtained without secondary dislocation of the fragments. The initial limitation of knee flexion is usually provided by a suitable knee orthesis, which can then be increased step wisely during the treatment course. 

Risks of conservative treatment include loss of full extension caused by nonunion of the fragments and stiffness of the knee, attributed to incongruity of the articular surface or prolonged immobilization.

Few recent studies are published demonstrating results after conservative treatment of nondisplaced fractures. These studies generally yield good to excellent results at given indications [12]. In Boström’s series, 219 of 422 patellar fractures were treated conservatively. 54% of these patients showed excellent results, 44% good results and only two failures occurred [9]. 

## Preoperative planning and surgical approach

Before surgical repair of the patella a brief preoperative planning should be obtained. This includes the choice of implants, surgical approach and a drawing of the fracture pattern with the estimated implant position. Thereby the surgeon gets acquainted with the fracture pattern and the required equipment can be chosen in advance. The procedure is performed under epidural or general anaesthesia with the patient placed in a supine position. Perioperative antibiotics should be administered approximately 30 minutes before skin incision. An intraoperative thorough physical examination – especially focussed on the ligamentous structures of the knee – should be performed prior to placing a tourniquet to the patient’s thigh. 

Of all surgical approaches described in literature, two approaches are currently favored to the patella. The transverse approach provides, besides satisfactory cosmetic results, good access to both the patella and the ligamentous structures of the extensor mechanism [60]. The longitudinal midline incision, which is used as standard approach in our center, allows excellent exposure to the patella and additionally does not interfere with approaches for subsequent implantation of a TKA. During surgical repair a small lateral parapatellar arthrotomy should be performed for direct visualization of the articular surface of the patella to control the reduction either by direct visualization or by palpation [22]. Notably, this approach is reported to have the potential to impair the vascular supply and may contribute to a tight postoperative lateral retinaculum, associated with consecutive patellar tilt, excessive lateral patellar pressures and a higher rate of anterior knee pain [28], [47]. 

## Operative treatment

### Modified tension band wring

**Figure 3:**
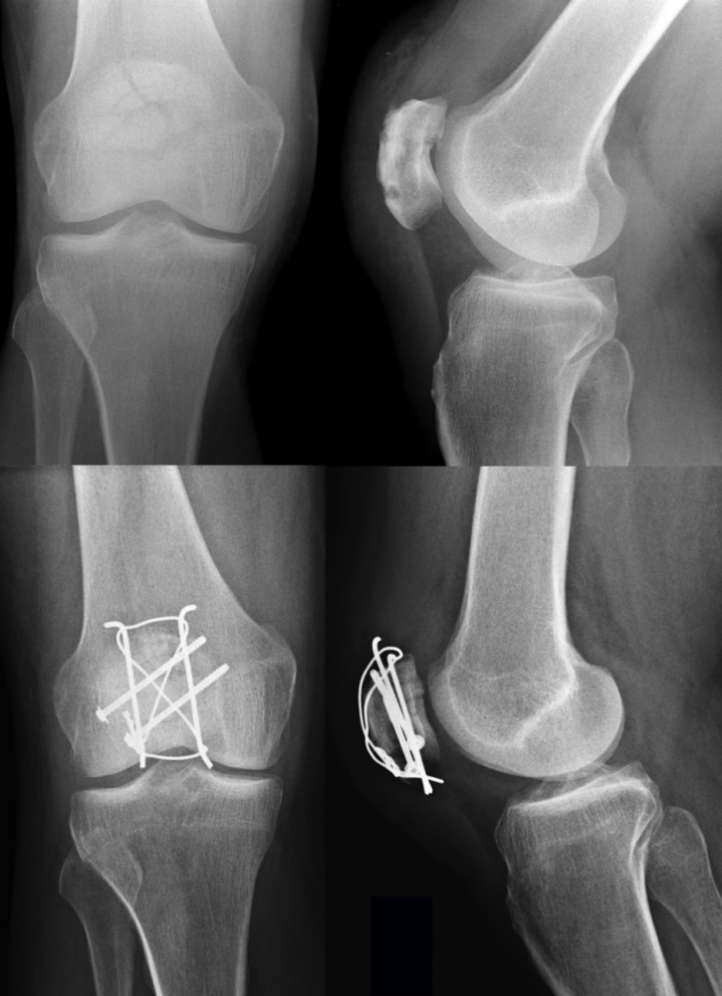
Preoperative X-rays of a comminuted patella fracture (above). 3 months post-surgery using a combination out of K-wires, screws, and an eight-shaped cerclage wiring (below).

As previously mentioned, every unstable fracture of the patella requires operative intervention. The modified tension band wiring, according to AO principles, is the most accepted and widely used technique for the treatment of displaced fractures of the patella, although several other techniques involving combinations of fixation techniques (i.e. K-wires, screws and cerclage wiring; Figure 3 (Fig. 3)) have been published [8], [60]. From a biomechanical point of view, the surgical procedure aims to neutralize tension forces applied to the patella via the extensor mechanism and convert them into compression forces. For this purpose, at least two K-wires are placed perpendicular to the fracture line and a tension band is applied in an eight-shaped manner to secure reduction. The ends of the K-wires are then twisted and buried in the patella.

An additional circular cerclage can be placed around the equator of the patella in order to increase the stability of the osteosynthetic construct. In selected cases with complex fracture patterns or distinct fractures to the distal pole of the patella application of a transosseous patellotibial cerclage, as described by McLaughlin, is recommended [56]. This helps to secure the reduction by decreasing the tension forces which are exerted by the quadrizeps muscle during knee flection. Despite this internal fixation, an additional immobilization in full extension and solely passive mobilization of knee flexion for 4–6 weeks is required in those cases [73]. One of the major risks of this method remains the overtightening of the cerclage resulting in a patella baja. To ensure the correct length of the cerclage an image intensifier should be used intraoperatively to assess and compare the distance of the tibial tuberosity and the distal pole of the patella of the contralateral knee. Furthermore, this cerclage has to be removed at 6 weeks after surgery and implant failure during the patient’s course is seen frequently. After stabilizing the patella itself, injuries to the extensor mechanism are repaired, if necessary. 

Biomechanical studies have demonstrated that an eight shaped tension band technique provides stability superior to circular wiring, but also implies a higher risk of soft tissue irritation due to prominence of the hardware [45]. It is imperative to position the tension band in close proximity to the bone, to minimize secondary dislocation of the fragments [63].

Although monofilament stainless steel wires are mainly used for tension band wiring, braided, non-absorbable polyester sutures (Ethibond-Ethicon Ltd., Edinburgh) have shown to have distinct advantages [27]. Biomechanical studies have shown that the braided sutures account for comparable results to steel wires [71]. Two of the main advantages of the suture technique are on the one hand a better stress distribution due to the flexibility of the braided wire. On the other hand, a closer proximity of the suture to the bone can be achieved more easily compared to a conventional wire. Furthermore, the intraoperative application of the braided suture is regarded to be easier and the rate of secondary hard ware removal is considerably lower than using classic stainless steel wires [27].

### Screw fixation with modified tension band

**Figure 4:**
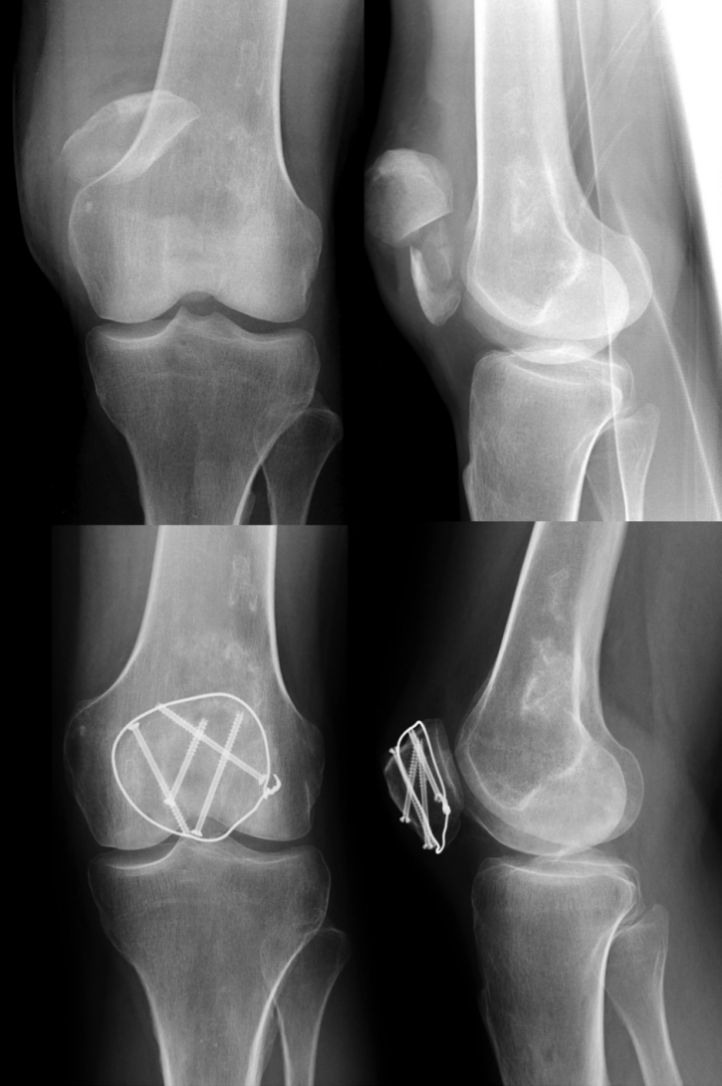
Preoperative X-rays of a comminuted patella fracture (above). 3 months post-surgery using screw fixation and modified tension band (below).

Screw fixation may decrease the risk of fragment dislocation as a result of tension band laxity and serves as a longitudinal stabilization of the fracture (Figure 4 (Fig. 4)). Screws should be applied perpendicular to the fracture line and fit the size of patellar bone (e.g. 3.5 mm cortical screws). 

Additional smaller fragments can be reattached to the main fragments using supplementary 2.4 or 2.0 mm screws tailored to the size of the fragments. If the fracture is fixed through a lag screw fixation an additional circular cerclage should be applied either using conventional stainless steel wires or braided sutures. Thelen et al. were able to show in a biomechanical analysis, that cannulated screws with anterior tension wiring is significantly superior to conventional modified anterior tension wiring in terms of preventing fracture displacement [79].

As a variant, the cerclage wire (1.2 mm) is passed close to the base of the patella and behind the screws as well as through a transverse channel of the distal pole [45], [60]. 

Berg published a technique in which the cerclage wire is passed through two parallel cannulated screws. He stated that advantages include a low-profile construct with minimal hardware irritation and that this technique can maintain anatomic reduction even in osteoporotic bone [6]. Carpenter et al., who compared three different fixation techniques, have confirmed these initial findings. In this study single screw fixation was compared to a tension band/K-wire construct as well as a modified tension band which was applied through cannulated screws according to the above mentioned technique. The highest load to failure was found for a gauge wire, passed through a cannulated lag screw to function as a modified tension band [15]. This method is technically demanding and data show, that it should not be used in comminuted fractures. However, this technique is considered to offer the ability of early rehabilitation with little hardware irritation while decreasing the risk for loss of reduction and has become the golden standard for simple transverse fractures of the patella [12], [14], [15].

**Figure 5:**
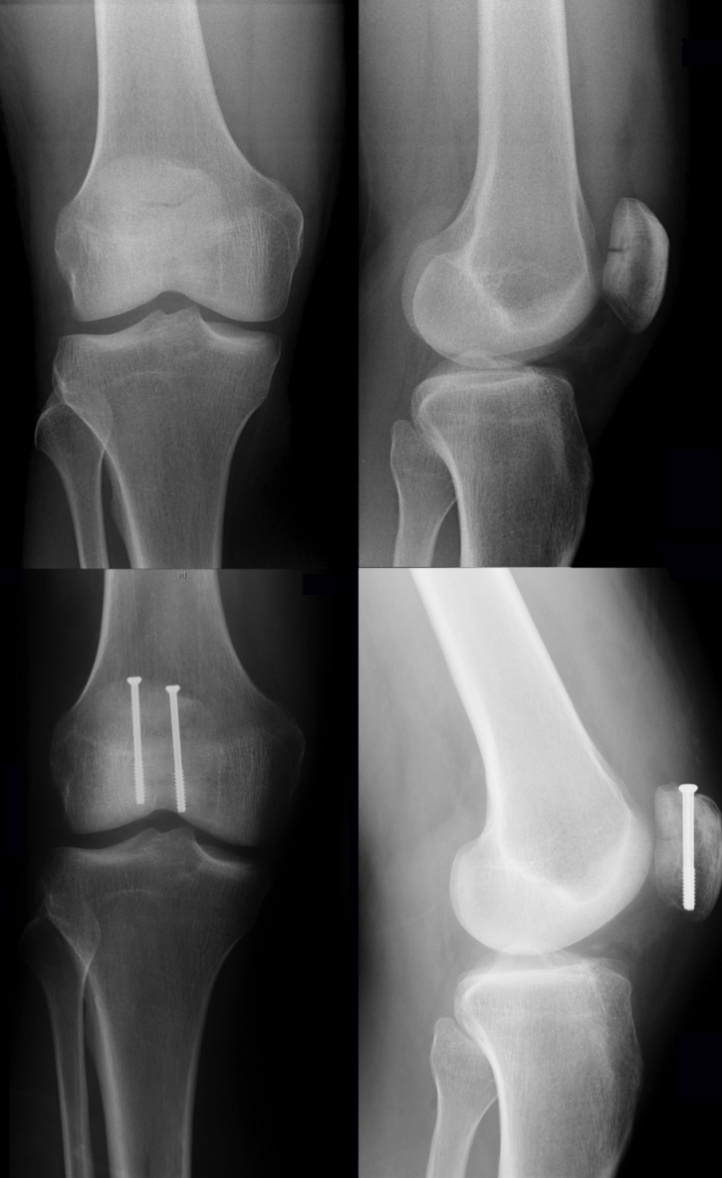
Preoperative X-rays of a non-comminuted transverse patella fracture (above). 3 months post-surgery using percutaneous screw fixation (below).

For simple transverse or vertical fractures in patients with adequate bone stock, lag screw fixation can also be considered without tension band wiring to prevent unnecessary dissection of surrounding soft tissue and thus preserving the vascular supply (Figure 5 (Fig. 5)). 

Subsequently, percutaneous techniques for modified tension band and cannulated screw fixation have been described [17], [55]. In selected cases when anatomic reduction can be achieved in a closed technique, cannulated screw insertion can be of advantage because it preserves the vascular supply of the patellar fragments and the risk for postoperative adhesions causing post-operative contracture is reduced. In the presence of severe soft tissue damage, a closed reduction and percutaneous fixation technique may be considered as long as sufficient reduction can be achieved.

### Partial patellectomy

In some cases, the fracture of the patella consists of one main proximal fragment and a severely comminuted lower pole of the patella. In this condition sufficient reduction and stable fixation is unlikely to be achieved and partial patellectomy has to be considered [67]. However, partial resection of the lower pole reduces the distance between the patella and tibial tuberosity and can lead to a patella baja with increased patellofemoral contact pressures leading to higher rates of anterior knee pain as well as an early onset of osteoarthritis [45]. In order to maintain the functional integrity of the extensor mechanism the central part of the patella and a total of two-thirds of the articular surface have to be preserved. Because of the powerful tension, which is developed by the quadriceps muscle complex, significant stress is applied on the reconstruction of the lower patellar pole. A temporary McLaughlin cerclage for the first 6 weeks is recommended for stress protection and prevention of re-dislocation.

### Patellectomy

Preservation of the patella is deemed necessary to preserve the extensor mechanism. In 1909 Heideck reviewed 1,100 patellar fractures and stated that total patellectomy should be considered on a case-by-case basis only as a last resort if the patella is not salvageable [34]. Several subsequent studies confirmed this finding and concluded that the clinical results after patellectomy are devastating [59], [82]. Kaufer et al. reported that an increase of quadriceps muscle force of up to 30% is necessary in order to sufficiently extend the knee after patellectomy. In addition, traction forces of the quadriceps muscle and the patella tendon increase by 15–50% after patellectomy [40], [41]. This usually results in persistent anterior knee pain, restricted range of motion, episodes of giving way, swelling and significant reduction of quadriceps strength [50]. Today, the biomechanical role and importance of the patella is acknowledged and thus every attempt is made to avoid patellectomy [47]. Against this background, it should only be considered as a salvage procedure in severe cases of osteomyelitis or severe comminuted fracture pattern. Even for severely displaced and comminuted fractures any effort of reconstruction should be made. 

### Osteochondral fractures of the patella

Chondral or osteochondral fractures usually occur during an acute dislocation of the patella as mentioned above. In case of dislocated osteochondral fragments, operative treatment is indicated. Depending on the size, location and vitality of the fragment several surgical techniques and options can be assessed. However, cartilage repair of the patella remains challenging and provides less satisfactory results when compared with the femoral condyles [32], [39], [46].

To date, arthroscopic techniques are well established and regarded as the method of choice. Reported management includes arthroscopic removal, marrow-stimulating techniques such as microfracture, mosaicplasty, immediate refixation of large osteochondral fragments and autologous chondrocyte implantation (ACI) [29], [31], [44], [49], [53]. 

Microfracture of the patella has been shown to lead only to a temporary improvement, with secondary deterioration after 18 to 36 months [46]. Mosaicplasty with the use of osteochondral autograft transfer has been demonstrated to result in very inconsistent results [4], [32]. This is attributed to the mismatch in cartilage thickness between the donor and recipient sites [81]. Gomoll et al. evaluated a total of 110 patients with cartilage defects of the patella after treatment with ACI. Patients improved significantly in clinical function as well as patient satisfaction represented by more than 90% of patients willing to undergo ACI again. Only 8% of patients were considered as treatment failures [26]. These results are in line with those of other studies of patellofemoral ACI [24], [62].

### Open fractures

The treatment of open fractures of the patella follows the same algorithm as in open long bone fracture care [74]. These cases require urgent intervention in order to avoid osteomyelitis and septic arthritis. Depending on the degree of soft tissue damage and the overall situation of the patient (mono trauma versus multiple injured patient) the treatment follows a staged concept. Débridement, irrigation, appropriate antibiotics and a stable fixation are the principles of treatment. As an external fixation of the patella is neither feasible nor advised, the use of a knee spanning external fixator can be indicated depending on the accompanying soft tissue damage. All attempts should be made to achieve a sufficient and closed soft tissue envelope. Temporary artificial skin substitutes or vacuum assisted closure techniques should be balanced after thorough débridement. Even though open patellar fractures are mostly associated with a high energy trauma and a significantly higher ISS (Injury Severity Score) as compared to patients with closed fractures, comparable results can be achieved [1].

### Clinical outcome

There is a paucity of literature reporting about the clinical and radiological outcome of patellar fractures, as outcome parameters vary considerably. Although predictable union rates have been achieved, little is known about the functional outcome, quality of life and lower extremity function. 

Nevertheless, general consensus is reached that non- or minimally displaced, transverse fractures, which have been treated conservatively yield the best outcome. Vice versa, comminuted fractures of the patella have the worst outcome resulting in 30–50% of the patients suffering from persistent anterior knee pain and 15–30% from functional impairment [51]. More recently, Lazaro et al. conducted a study of 30 patients who suffered from a fracture of the patella and were treated by open reduction and internal fixation. Despite radiographic bone healing, nearly anatomical reduction, reliable reconstruction of the extensor mechanism and physiotherapy, 80% of the patients reported about anterior knee pain [47]. 

These findings are consistent with long-term results of LeBrun et al., who reported that patients have persistent physical sequelae at a median of 6.5 years post injury, as reflected by poor SF-36 and KOOS (Knee Injury and Osteoarthritis Outcome) score outcomes. Hardware removal was required in 52% of patients, whereas 28% of those with retained fixation reported about implant-related pain. The authors also noted a restricted range of motion of more than 5° of extension lack in 15% and a lack of flexion in 38%. Isometric dynamometric testing revealed a mean extension deficit of 26% and a loss of extension power of 30% when compared to the contralateral side. Although not statistically significant, there was a trend towards better results for screw fixation in combination with tension band wiring [48].

## Complications

### Loss of knee motion

The most common complication after patellar fractures is a decreased range of motion (ROM). Infections, prolonged postoperative immobilization and improper rehabilitation are reported to be the main reasons. Subsequently, the traumatic as well as the iatrogenic soft tissue damage can lead to subcutaneous and intraarticular adhesions resulting in the same problem. Nikiforidis et al. demonstrated that in most cases only the terminal degrees of flexion are affected which is usually well tolerated by the patients [63]. Conservative treatment including intense physiotherapy is the standard method in these cases. If flexion is still decreased after the conservative approach, partial or total hardware removal in combination with an arthroscopic arthrolysis of intra-articular adhesions should be contemplated [42]. If an arthroscopic procedure is planned, both a sciatic and femoral nerve block should be applied perioperative in order to prevent pain-related restriction of postoperative motion.

Starting from day one after arthroscopic arthrolysis, the knee joint is mobilized using a continuous passive motion (CPM) device and extensive physiotherapy is applied in order to obtain a normal range of motion. 

Even though quadricepsplasty has been advocated in the past, it implies a high intraoperative risk and a longstanding rehabilitation and immobilization of the knee joint itself [2], [20], [30]. 

### Loss of reduction

**Figure 6:**
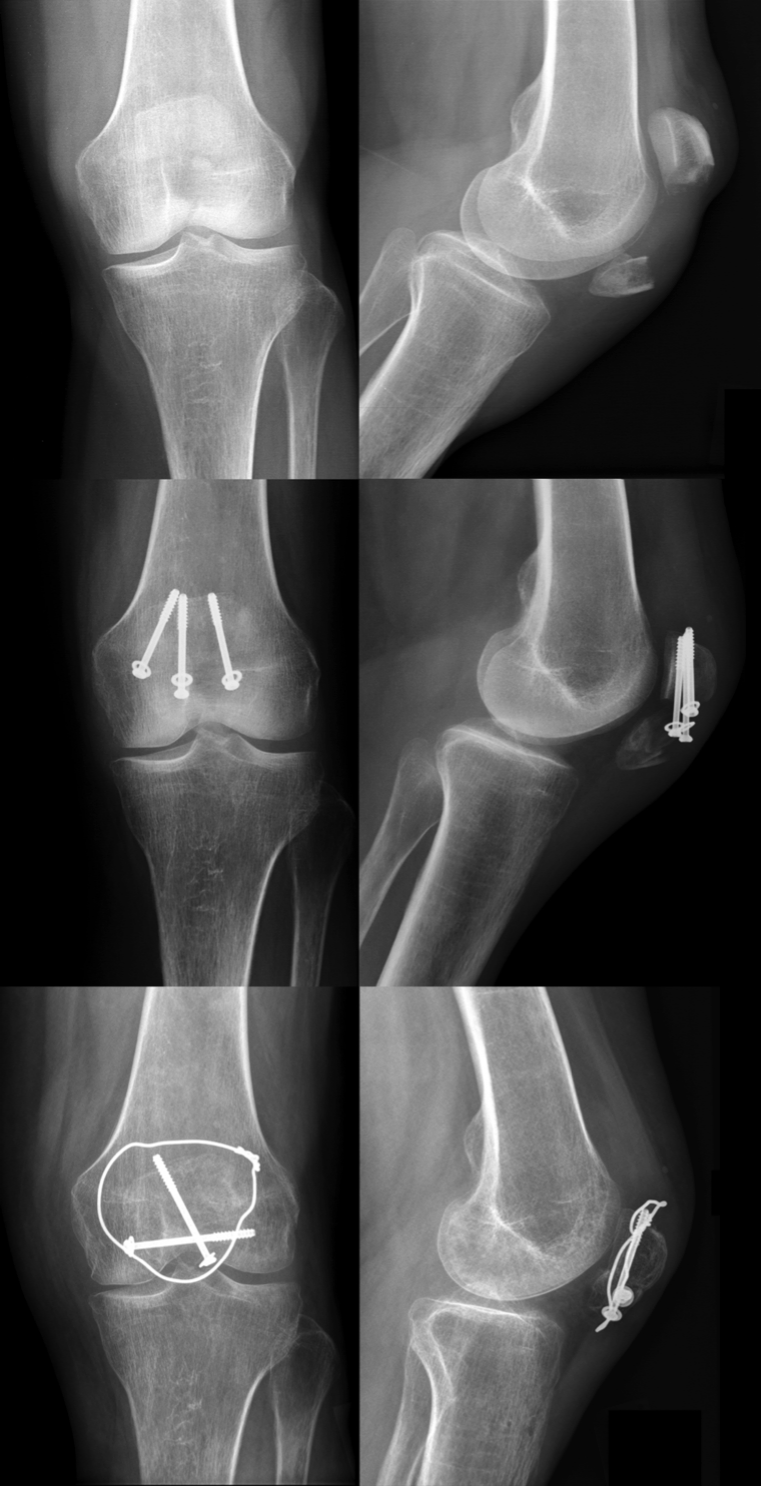
Preoperative X-rays of a transverse patella fracture (above). Loss of reduction with screw pullout 3 month after surgery (middle). 3 months after revision surgery with screw fixation and additional modified tension band (below).

Loss of reduction occurs in up to 20% of operatively treated patellar fractures (Figure 6 (Fig. 6)) [9], [10], [63], [76]. This is usually attributed to technical errors, most commonly involving improper placement or tensioning of the modified tension band, consecutively causing construct instability and fracture displacement [37], [76]. 

Miller et al. were able to show that the use of K-wires with or without tension band correlates with higher failure rates, compared with the use of screws [58]. The indication for revision surgery is given if the fragments separate more than 3 mm from each other or if the articular surface presents with an incongruity of more than 2 mm [10].

### Infections

The subcutaneous position of the patella makes it prone to open injuries and subsequent infection. Reported infection rates range from 3 to 10% [19], [63]. Both, compromised soft tissue due to the injury and hardware irritation – especially over the twisted portion of the tension band on the anterior subcutaneous surface of the patella – are suggested to be factors leading to a higher risk for infection [76]. Infections should be treated by standard protocol including irrigation, debridement, resection of necrotic soft tissue and application of culture-specific antibiotics. In the rare case of severe osteomyelitis of the patella which fails to be operatively managed, patellectomy can be discussed as an exceptional salvage procedure.

### Delayed or nonunion

The incidence of nonunion or delayed union of patella fractures ranges from 2.7 to 12.5% [43], [61], [80]. A strong correlation could be demonstrated between open fractures, transverse fractures and immobilization during conservative treatment and nonunion [43], [61], [80]. A treatment protocol for non unions of the patella does not exist and little is known about the results of different approaches. The treatment principles of non unions of long bone reflected by the diamond concept should be applied also to these cases in order to achieve bony union. The optimal treatment concept of nonunions has to comprise the clinical situation as well as patient based factors (age, comorbidities and demand) in order to find the best treatment strategy and in some cases a conservative approach may be the best option. Patients with low demands tend to adapt a gait pattern in full extension and internal rotation of the lower limb and do not require surgical intervention [61]. Vice versa, patients who perform heavy physical work or participate in sports usually require revision surgery [23].

### Patellafemoral osteoarthritis

The development of patellofemoral osteoarthritis after fracture of the patella is considered at approximately 8.5% [57]. The initial injury-related damage to the articular cartilage is advocated to be the determining factor leading to degenerative changes. Chronically increased contact loads and decreased mechanical resistance due to impaired nutritive supply may accelerate this degeneration [47]. The incidence of patellofemoral osteoarthritis is difficult to ascertain from the literature and only few studies are available with rather controversial results. Sorensen et al. stated that the incidence of osteoarthritis following a fracture of the patella is not dependent on the treatment (conservative versus operative) [77]. Boström et al. noted that the development of osteoarthritis depends on the initial cartilage damage in first place and second to the quality of reduction. He was able to show that a step in the articular cartilage of more than 1 mm leads to higher rates of posttraumatic osteoarthritis [10]. Consequently, the treatment method chosen should have a focus on reduction of the articular surface in order to avoid the early onset of posttraumatic osteoarthritis because there no sufficient clinical solutions exist to treat a patella femoral osteoarthritis exclusively. These patients often result in a total knee arthroplasty at young age. 

### Hardware removal

Lazaro et al. reported a rate of 37% hardware removal due to prominent and symptomatic implants as a result of breakage or continuous soft tissue irritation [47]. Other authors report a hardware removal rate as high as 10% within the first 12 months [37]. Symptomatic implants are reported twice as frequent in K-wires in comparison to cannulated screws [35]. Attention to detail in performing tension band wiring may reduce the number of symptomatic hardware, as most of the soft tissue irritation comes from broken tension bands or bulky wire knots.

## Notes

### Competing interests

The authors declare that they have no competing interests.

### Authorship

The authors Gwinner C and Märdian S attributed equally to this article.
